# Multimodal CT radiomics predicts PD-1 inhibitor efficacy in advanced gastric cancer: a two-center validation study

**DOI:** 10.1186/s13244-025-02096-1

**Published:** 2025-10-31

**Authors:** Zhipeng Wang, Yinchao Ma, Jiahe Tan, Ming Li, Chenyang Qiu, Kun Han, Shuzhen Wu, Haiyan Wang

**Affiliations:** 1 Department of Radiology, Shandong Provincial Hospital Affiliated to Shandong First Medical University, 250021 Jinan, China; 2 School of Radiology, Shandong First Medica University, Taian, Shandong China; 3 University of California, Davis, CA USA

**Keywords:** Gastric cancer, Programmed cell death 1 inhibitors, Radiomics, Chemo-immunotherapy

## Abstract

**Objectives:**

In this study, we developed a multi-modal CT-based machine learning model to predict the response of gastric cancer (GC) patients to first-line chemotherapy combined with PD-1 inhibitors and performed external validation and multi-model comparisons.

**Materials and methods:**

We retrospectively analyzed the clinical data of 348 patients with GC who underwent immunotherapy. The patients were categorized into an internal validation cohort (center A, *n* = 272) and an external validation cohort (center B, *n* = 76). Pre-treatment clinical and CT radiomics features were extracted to develop three models: a clinical model, a radiomics model and a clinical-radiomics model. The classifiers included logistic regression (LR), linear support vector classification (Linear SVC), support vector machine, and random forest.

**Results:**

A total of 19 radiomics signatures and 5 clinical feature signatures were selected. In the radiomics model, the Linear SVC algorithm achieved an area under the receiver operating characteristic curve (AUC) of 0.88 and 0.76 in internal and external validation sets, respectively. In both the clinical model and the clinical-radiomics model, the LR algorithm demonstrated high and stable predictive performance in the internal (AUC = 0.89 and 0.94) and external validation datasets (AUC = 0.76 and 0.85). Among all models in the external validation dataset, the clinical-radiomics model utilizing LR outperformed all other classifiers.

**Conclusions:**

The clinical-radiomics model, in combination with the LR algorithm, provides a reliable and effective method for predicting the early response of advanced GC patients treated with programmed cell death-1 (PD-1) inhibitors combined with chemotherapy.

**Critical relevance statement:**

CT radiomics and laboratory parameters were used to evaluate early prediction of response to PD-1 inhibitors combined with chemotherapy in patients with advanced gastric cancer. This clinical-radiomics model provides a novel approach to predict immunotherapy efficacy and prognosis.

**Key Points:**

- Evaluating the efficacy of PD-1 inhibitors combined with chemotherapy in advanced gastric cancer using only clinical data is limited.
- Only some patients with advanced gastric cancer treated with the PD-1 inhibitors combined with chemotherapy achieved complete regression.
- This clinical-radiomics model showed good performance for predicting gastric cancer response to chemotherapy combined with PD-1 inhibitors.

**Graphical Abstract:**

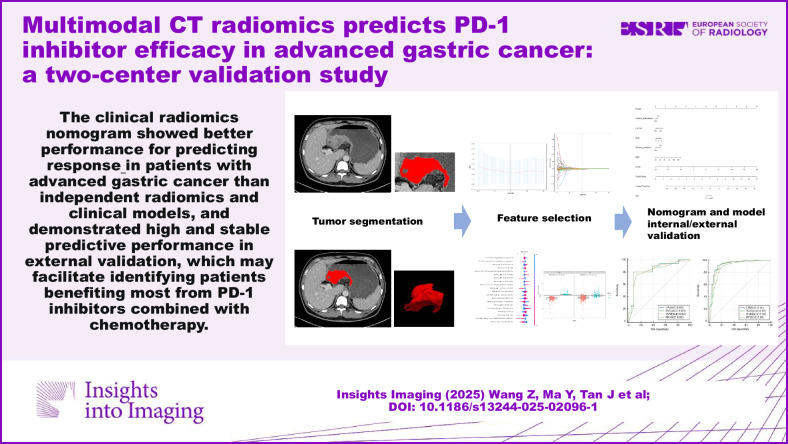

## Introduction

Gastric cancer (GC) is the fifth most common malignant tumor and the fifth leading cause of cancer-related death in the world [1], yet it is still a challenge to human health. Due to its insidious onset, atypical symptoms, and rapid progression, early-stage GC is often diagnosed at an advanced stage when they are treated [2].

In recent years, the rise of immunotherapy has revolutionized the landscape of cancer treatment, especially with the emergence of immune checkpoint inhibitors (ICIs) represented by anti-programmed cell death-1 (anti-PD-1) or anti-programmed cell death ligand-1 (anti-PD-L1). These therapies have led to significant advancements in the management of various malignancies, including advanced gastric cancer (AGC) [3]. At present, anti-PD-1 inhibitors have been officially approved for clinical application in a variety of cancers [4], and in current clinical application, chemotherapy combined with immunotherapy has become a primary treatment strategy for patients with AGC.

Although PD-1 inhibitors combined with chemotherapy have demonstrated superior efficacy over single chemotherapy in some patients with AGC [5], there are still some patients who do not benefit from it [6]. These individuals may experience disease progression, potential immune-related adverse events [7], and substantial healthcare costs. Therefore, there is an urgent need for simple and reliable predictors to identify patients who are likely to benefit from combination therapy. Existing predictive biomarkers, such as programmed death-ligand 1 (PD-L1) expression, tumor mutation burden (TMB), microsatellite instability-high (MSI-H), and mismatch repair (MMR) status, determine whether AGC patients are suitable for immunotherapy [8–11]. However, these biomarkers typically require biopsy acquisition, which is invasive and costly. In contrast, the acquisition of clinical laboratory tests and imaging features offers a simpler, non-invasive alternative. Moreover, due to tumor heterogeneity [12], biopsy samples may not fully reflect the overall characteristics of the tumor, while imaging examinations can reflect the overall characteristics of the tumor at a macroscopic level. Previously, Wang et al [13] found a correlation between changes in certain CT imaging features and pathological responses in GC patients receiving chemotherapy. Additionally, several studies have explored the relationship between peripheral blood indicators or their derived innovative indicators and the efficacy of immunotherapy in GC patients [14, 15]. However, most studies have only revealed the association between predictors and clinical outcomes, and there is still a lack of joint assessment models or comprehensive prediction models established with multiple biomarkers.

As an emerging field, radiomics is regarded as a non-invasive digital biopsy technology, which can extract quantitative image features from medical images in a high-throughput manner. These features can be converted into analyzable quantitative data, providing deeper insights into tumor microenvironment characteristics and heterogeneity [16–18]. Some previous studies have demonstrated that image features extracted by radiomics can be used as biomarkers to predict the efficacy of immunotherapy, which can predict the immunotherapy response of many cancers, including GC [19–21]. However, most of these studies lack external validation, and the generalization ability of radiomics model is difficult to be verified [22]. In addition, variations in modeling algorithms can affect the prediction performance of the model [23].

Therefore, this study developed and validated non-invasive imaging biomarkers using radiomics. By integrating clinical features and CT imaging features before treatment of GC patients, we modeled and compared the ability of different algorithmic models to predict the response to PD-1 inhibitors combined with chemotherapy. Identifying the most effective model could aid in the early stratification of patients likely to benefit from combination therapy, facilitating personalized treatment strategies. This can be helpful for early stratification of the benefits of combination therapy and individualized medical management.

## Materials and methods

### Patient inclusion and exclusion criteria

The study was approved by the Ethics Committee of Shandong Provincial Hospital (Ethics number: NSFC: NO.2022-402). Informed consent was waived because of the retrospective nature of the study.

With the approval of the local institutional ethics committee, Patients with AGC were treated with anti-PD-1 inhibitors combined with chemotherapy in two centers (center A: Shandong Provincial Hospital; center B: Shandong Cancer Hospital).

**Fig. 1:**
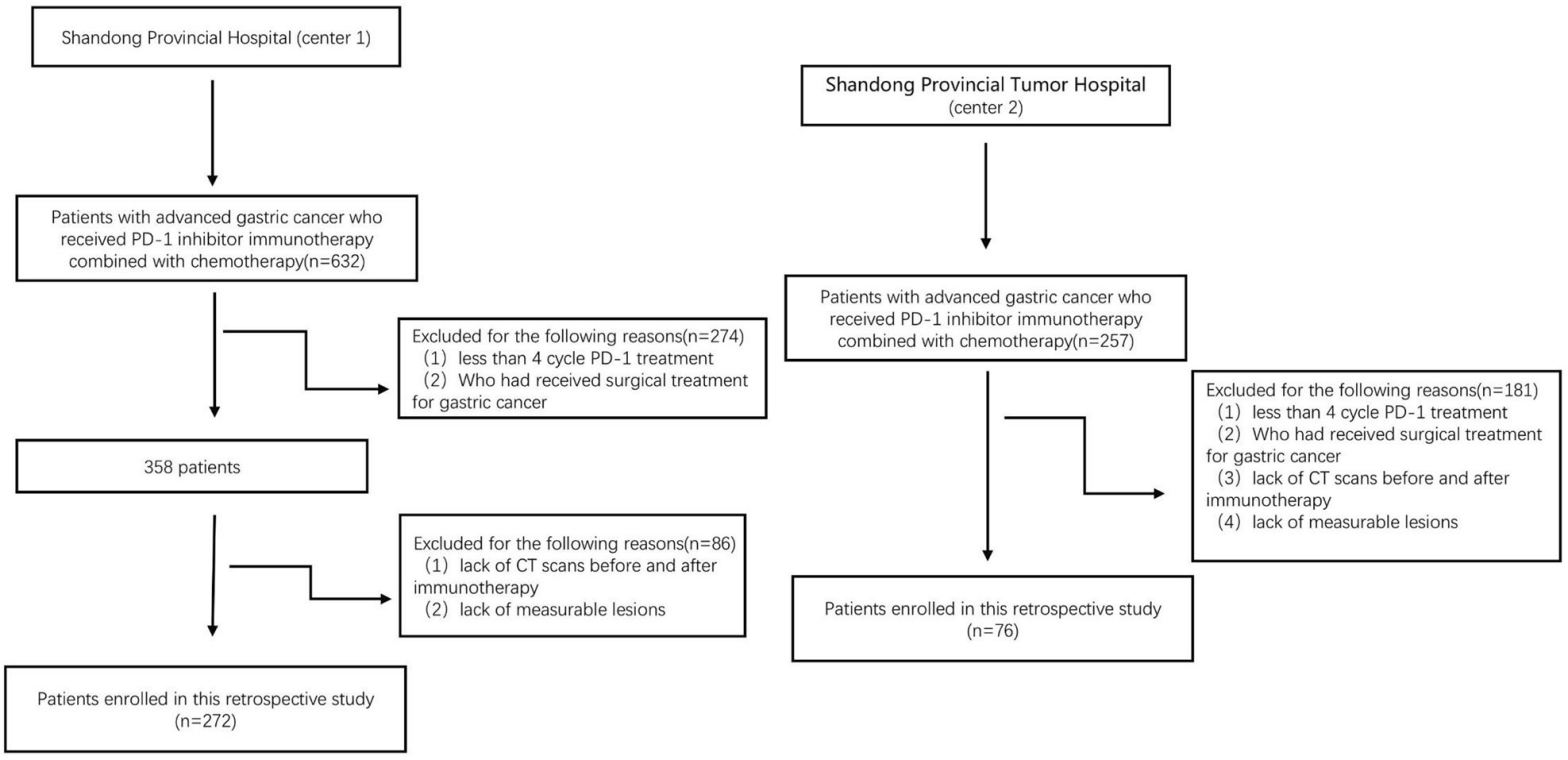
Shows the participant selection flow chart

The inclusion criteria: (1) Gastric cancer confirmed by pathology; (2) concurrent administration of at least four cycles of anti-PD-1 therapy plus chemotherapy. (3) At least one measurable lesion met the Response Evaluation Criteria in Solid Tumors (RECIST) version 1.1. (4) Complete CT imaging data before and after anti-PD-1 combined chemotherapy were available. Exclusion criteria were as follows: (1) gastric surgery before or during PD-1 inhibitor combined chemotherapy. (2) inadequate gastric dilatation hampered the measurement of the lesion. (3) incomplete clinicopathological data. Figure 1 shows the participant enrollment flow chart.

### Treatments and study assessments

In this study, patients received three-week cycles of PD-1 inhibitors, including camrelizumab, sintilimab, nivolumab, tislelizumab, and pembrolizumab, along with first-line standard chemotherapy. The main chemotherapy regimens included SOX (S-1 + oxaliplatin), XELOX (capecitabine plus oxaliplatin) and FOLFOX (leucovorin, fluorouracil, and oxaliplatin). The response to PD-1 inhibitors combined with chemotherapy was evaluated by a radiologist using a CT scan approximately 4 weeks after the completion of four cycles of PD-1 inhibitors combined with chemotherapy. The results were grouped according to the RECIST 1.1. Outcomes included complete response (CR), partial response (PR), stable disease (SD), and progressive disease (PD), and all patients were classified as non-PD responders (CR, PR, or SD) and PD responders.

The CT imaging images of patients taken within two weeks before their first PD-1 inhibitor combination chemotherapy and after four cycles of treatment were obtained through the Picture Archiving and Communication System (PACS). Baseline clinical data (collected ≤ 2 weeks before treatment initiation) were extracted from electronic medical records, including gender, age, body mass index (BMI), treatment regimen, presence or absence of distant metastasis and laboratory indicators. Laboratory measurements were collected during the 2 weeks before the first treatment with a PD-1 inhibitor combined with chemotherapy, and clinical information is recorded in Table 1.

**Table 1:** Baseline characteristics of the Center A cohort and the Center B cohort

	Center A cohort (*N* = 272)			Center B cohort (*N* = 76)		
	PD responders (*N* = 66)	non-PD responders (*N* = 206)	*p*-value	PD responders (*N* = 22)	non-PD responders (*N* = 54)	*p*-value
Age (%)			0.456			0.819
≤ 60	31 (47.0)	86 (41.7)		10 (45.5)	23 (42.6)	
> 60	35 (53.0)	120 (58.3)		12 (54.5)	31 (57.4)	
Gender (%)			0.224			0.770
Male	45 (68.2)	156 (75.7)		17 (77.3)	40 (74.1)	
Female	21 (31.8)	50 (24.3)		5 (22.7)	14 (25.9)	
Immunotherapy regimen (%)			0.67			0.729
Camrelizumab	36 (54.5)	91 (44.2)		11 (50.0)	25 (46.3)	
Sintilimab	23 (34.8)	88 (42.7)		10 (45.5)	24 (44.4)	
Nivolumab	3 (4.5)	11 (5.3)		0 (0.0)	3 (5.6)	
Tislelizumab	3 (4.5)	10 (4.9)		1 (4.5)	2 (3.7)	
Pembrolizumab	1 (1.5)	6 (2.9)		0	0	
Tumor location (%)			0.897			0.847
Cardia	22 (33.3)	74 (35.9)		8 (36.4)	19 (35.2)	
Body	24 (36.4)	69 (33.5)		10 (45.5)	22 (40.7)	
Antrum	20 (30.3)	63 (30.6)		4 (18.2)	13 (24.1)	
Lymph nodes with a short diameter > 1 cm (%)			0.898			0.77
Absence	19 (28.8)	61 (29.6)		6 (27.3)	13 (24.1)	
Presen	47 (71.2)	145 (70.4)		16 (72.7)	41 (75.9)	
Obscuration of the perigastric fat space (%)			0.415			0.935
Absence	9 (13.6)	37 (18.0)		4 (18.2)	12 (22.2)	
Presen	57 (86.4)	169 (82.0)		18 (81.8)	42 (77.8)	
Serosal invasion (%)			0.002			0.387
Absence	3 (4.5)	43 (20.9)		1 (4.5)	8 (14.8)	
Presen	63 (95.5)	163 (79.1)		21 (95.5)	46 (85.2)	
Distant metastasis (%)			< 0.001			0.002
Absence	19 (28.8)	145 (70.4)		4 (18.2)	31 (57.4)	
Presen	47 (71.2)	61 (29.6)		18 (81.8)	23 (42.6)	
BMI	23.22 (21.88, 23.73)	23.26 (21.36, 24.61)	0.663	23.23 ± 3.04	23.07 ± 3.28	0.843
Maximum tumor thickness (cm)	2.21 (1.50, 2.98)	2.10 (1.63, 2.70)	0.842	2.100 (1.825, 2.675)	2.200 (1.500–2.675)	0.566
Maximum tumor diameter (cm)	5.55 (4.03, 8.28)	5.850 (4.00, 7.68)	0.718	6.38 ± 2.40	5.99 ± 2.86	0.573
CT values in the unenhanced phase (HU)	36.850 (32.33, 39.10)	36.850 (33.10, 40.28)	0.843	31.250 (30.13, 35.40)	32.450 (28.73, 35.20)	0.714
CT values in the arterial phase (HU)	59.90 (47.50, 71.38)	67.350 (54.80, 77.15)	0.001	54.050 (49.58, 61.18)	55.300 (49.60, 62.68)	0.457
CT values in the venous phase (HU)	77.15 (67.73, 93.23)	77.300 (65.58, 88.85,)	0.419	66.750 (61.03, 76.20)	66.450 (60.63, 74.90)	0.909
CT values in the delayed phase (HU)	76.80 (67.10, 87.60)	76.050 (67.78, 87.83)	0.883	63.950 (60.50, 73.48)	69.950 (64.18, 77.90)	0.159
Tumor Arterial attenuation (HU)	21.55 (14.30, 31.48)	30.100 (21.33, 37.98)	< 0.001	20.350 (14.35, 28.80)	23.100 (18.53, 29.88)	0.186
Tumor venous attenuation on portal phase (HU)	40.25 (32.33, 55.00)	39.650 (31.45, 50.98)	0.533	35.200 (29.00, 45.25)	36.600 (27.13, 44.20)	0.766
AEF (%)	55.12 (45.66, 63.75)	71.700 (57.98, 83.61)	< 0.001	62.10 (51.75, 68.35)	70.740 (58.68, 75.28)	0.004
CEA (ng/mL)	7.96 (2.63, 16.85)	3.98 (1.96, 15.10)	0.037	13.77 (3.91, 48.63)	4.66 (2.00, 34.34)	0.162
AFP (ng/mL)	3.00 (1.90, 11.08)	2.50 (1.60, 6.80)	0.095	3.68 (2.33, 18.42)	3.67 (2.11, 9.26)	0.532
CA125 (U/mL)	25.17 (12.15, 77.75)	14.40 (8.87, 31.70)	0.001	51.79 (20.88, 187.25)	33.21 (18.75, 79.28)	0.236
CA199 (U/mL)	22.34 (12.15, 83.45)	16.95 (10.25, 43.75)	0.146	20.25 (16.60, 126.68)	22.10 (6.35, 110.13)	0.287
CA72-4 (U/mL)	14.90 (4.00, 140.50)	5.66 (2.30, 21.80)	0.001	16.20 (9.70, 32.33)	8.10 (3.53, 18.23)	0.052
PLT (10^9^/L)	223.00 (150.75, 276.75)	255.500 (199.00, 300.50)	0.037	259.27 ± 87.60	244.30 ± 91.64	0.515
lymphocyte (10^9^ /L)	1.26 (0.98, 1.71)	1.525 (1.20, 1.86)	0.003	1.29 (1.09, 1.71)	1.34 (1.12, 1.78)	0.433
Neutrophilcount (10^9^/L)	3.58 (2.67, 4.90)	3.26 (2.43, 4.34)	0.118	3.22 (2.81, 4.60)	3.77 (2.79, 4.94)	0.397
monocyte (10^9^/L)	0.50 (0.35, 0.65)	0.47 (0.36, 0.59)	0.72	0.51 (0.41, 0.68)	0.54 (0.42, 0.69)	0.868
SII	541.76 (350.56, 1044.94)	517.41 (323.15, 893.94)	0.302	657.72 (411.83, 970.80)	666.99 (361.34, 1008.44)	0.805
MLR	0.39 (0.29, 0.53)	0.32 (0.24, 0.43)	0.002	0.36 (0.30, 0.53)	0.41 (0.28, 0.50)	0.779
NLR	2.65 (1.86, 4.56)	2.05 (1.53, 3.15)	0.002	2.69 (2.08, 3.98)	2.79 (2.09, 4.01)	0.963

Two radiologists with more than 3 years of experience in abdominal diagnosis independently reviewed all CT images. CT image features included the following characteristics: (1) the maximum axial thickness and maximum axial length of the tumor, the main location (refer to endoscopic findings: cardia, body, and antrum), and whether the visceral peritoneum was invaded; (2) perigastric fat turbidity; Whether there are enlarged lymph nodes (defined as regional lymph nodes with a short diameter of more than 1 cm in the upper abdomen); (3) Parameters related to CT value: the maximum section of the tumor was taken on the axial view (avoiding necrosis and vascular areas as much as possible) and the CT value of the plain scan, arterial phase, venous phase, and delayed phase were measured respectively. The final result was the average of the measured data of the two doctors. The tumor arterial attenuation (HUA = HU_A_ − HU_U_), venous attenuation (HUV = HU_V _− HU_U_), and arterial enhancement fraction (AEF) = (HU_A_ − HU_U_)/(HU_V_ − HU_U_) ×  100% [19] (HUA represents arterial attenuation; HUV represents venous attenuation; HU_U_ represents the CT value of the lesion in plain scan phase. HU_A_ represented the CT value in the arterial phase. HU_V_ represents CT value in the venous phase [20].

In our study, continuous variables conforming to the normal distribution were expressed as mean ± standard deviation, and non-normally distributed continuous variables were expressed as median (interquartile range, IQR). Categorical variables were expressed as frequencies and percentages. For all tests, a two-sided *p* < 0.05 was considered statistically significant.

We performed univariate logistic regression (LR) analysis for each variable using clinical information and CT imaging features. Subsequently, the variables with *p* < 0.05 were integrated, and variables in each model were assessed for multicollinearity. Finally, indicators with *p* < 0.05 in both models were selected as variables in the clinical model.

### CT imaging and image segmentation

Image analysis was performed by two gastrointestinal radiologists with more than 3 years and more than 20 years of experience. Using ITK-SNAP software (RRID: SCR_017341; http://www.itksnap.org) we manually segmented the primary tumor area on the cross-sectional CT images of the venous phase of the enhanced CT and delineated the lesion area as a region of interest (ROI). Disagreements that existed in the assessment were reassessed after discussion to reach an agreement.

The CT scan protocol is shown in Appendix Table S1.

### Radiomics feature extraction and selection

The CT radiomics feature extraction was performed using the PyRadiomics toolkit (version 3.1.0). We normalized the CT images using a rescaling operation. The images were resampled at a voxel size of 1 × 1 × 3 mm^3^ and interpolated using the B-spline algorithm. All segmented images were subjected to wavelet filtering and Gaussian Laplace filtering. Subsequently, the image was discretized to a fixed bin width. A total of 1051 radiomics features were extracted from the original segmented images, including first-order statistics, shape descriptors, gray-level co-occurrence matrix, gray-level run-length matrix, gray-level size-region matrix, and gray-level dependence matrix. The extracted radiomics features were standardized by *z*-score. Intraclass correlation coefficients (ICC) were used to evaluate the intra-observer and inter-observer reproducibility. Radiomics features with ICC values above 0.75 were retained. for further analysis. The characteristic of 0.75. The least absolute shrinkage and selection operator (LASSO) was used to select features, which can better solve the problem of multicollinearity in the analysis. The features of the screening are presented in Table 2.

### Model establishment and verification

To evaluate the performance of machine learning methods in predicting the response to immunotherapy in locally AGC, we designed three models using clinical risk factors, radiomics features, and a clinical-radiomics model: clinical model, radiomics model, and clinical-radiomics model. Each model was modeled using four machine learning methods (random forest (RF), support vector machine (SVM), linear support vector classification (Linear SVC), logistic regression (LR)). We used 10-fold cross-validation and grid search at a ratio of 8:2 to find the best modeling hyperparameters for each model in the center A dataset. The optimal model (based on training set performance) was validated on internal and external cohorts (center B).

The area under the receiver operating characteristic (ROC) curve (AUC) was plotted to evaluate the discriminative prediction ability of each model. The DeLong’s test was used to compare the AUC values among the models.

**Fig. 2:**
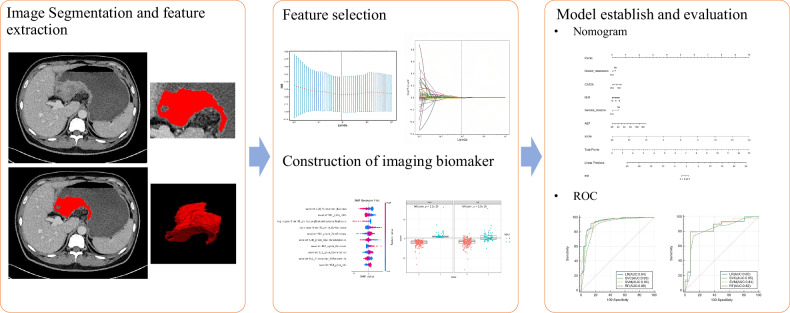
Flowchart of radiomics and clinical-radiomics model construction

We implemented all feature classification methods using the Python machine learning library (scikit-learn) and the R statistical platform (version 4.3.1). Figure 2 shows the workflow of radiomics analysis. MedCalc (version 22.21.0.0) was used for statistical analysis. Two-sided *p*-value of less than 0.05 were considered to indicate statistical significance. Statistical analyses were performed with SPSS (version 26).

## Results

A total of 348 patients were enrolled in the final analysis. Between June 2019 and June 2023, a total of 272 patients (206 non-PD responders and 66 PD responders) were recruited from center A, while 76 patients (54 non-PD responders and 22 PD responders) from Center B constituted the external validation cohort. The robustness of the model was tested on internal and external validation sets.

**Fig. 3:**
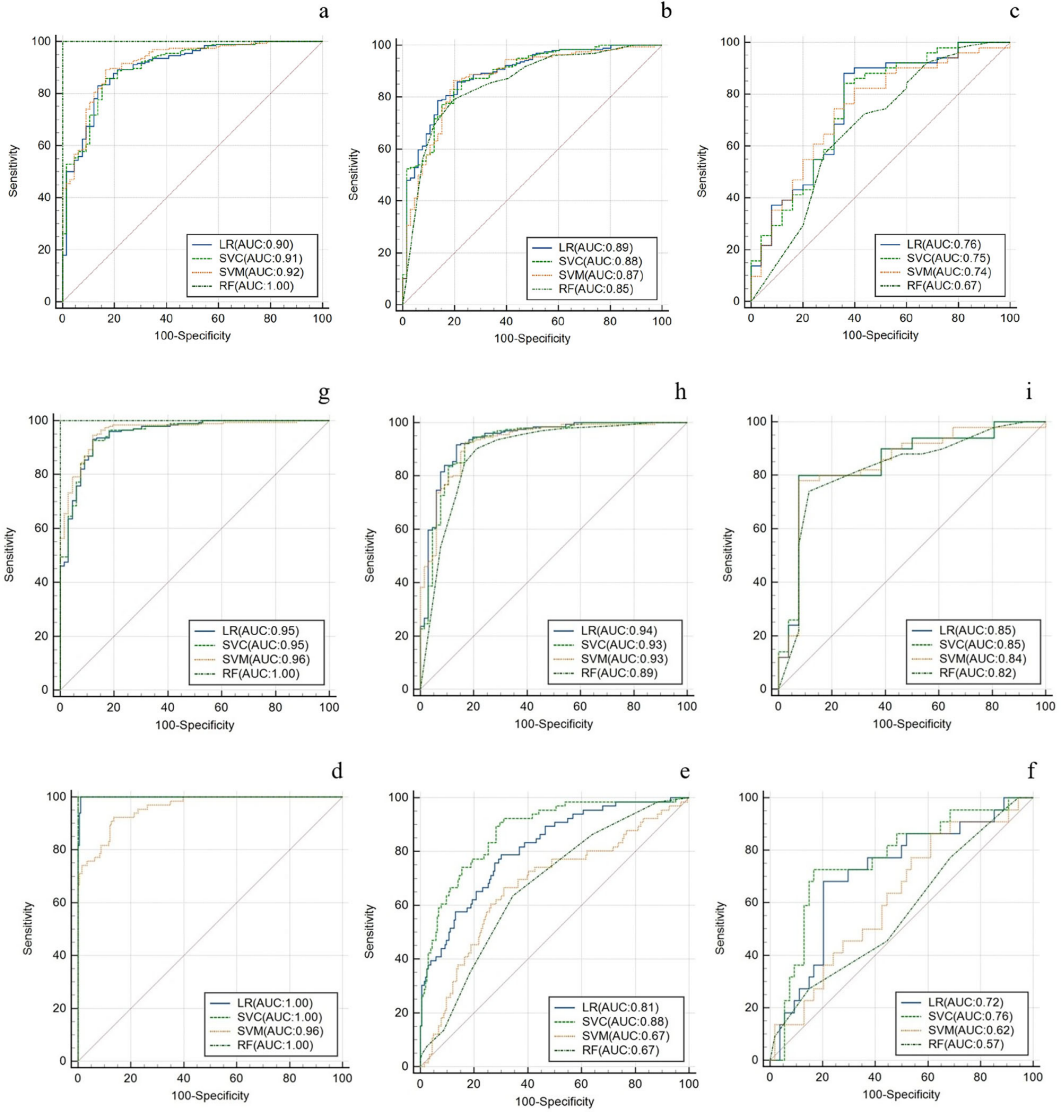
ROC curves of four classifiers in the clinical model for predicting pCR in the training (**a**), internal validation (**b**), and external validation sets (**c**). ROC curves of four classifiers in the radiomics model for predicting pCR in the training (**d**), internal validation (**e**), and external validation sets (**f**). ROC curves of four classifiers in the clinical radiomics model for predicting pCR in the training (**g**), internal validation (**h**), and external validation sets (**i**)

The clinical model was constructed using the *p*-value < 0.05 indicators of clinical information and CT imaging features, including neutrophil to lymphocyte ratio (NLR), carbohydrate antigen 72-4 (CA72-4), distant metastasis, serosal invasion (visceral peritoneum), and AEF. The AUCs of the clinical models based on RF, SVM, Linear SVC, and LR in the internal validation set were 0.85, 0.87, 0.88, and 0.89, respectively. The AUCs of RF, SVM, Linear SVC, and LR in the external validation set were 0.67, 0.74, 0.75, and 0.76, respectively (Table 2 and Fig. 3). The clinical model based on LR achieved the highest AUC in both the internal and external validation sets.

**Table 2:** The performance of clinical models, radiomics models and clinical-radiomics models in the Training cohort and External validation cohort

	Training set			Internal validation set			External validation set		
	AUC [95% CI]	Sensitivity	Specificity	AUC [95% CI]	Sensitivity	Specificity	AUC [95% CI]	Sensitivity	Specificity
Clinical classifier									
LR	0.90 [0.86–0.95]	0.79	0.86	0.89 [0.84–0.93]	0.79	0.83	0.76 [0.63–0.88]	0.69	0.67
SVC	0.91 [0.86–0.95]	0.80	0.85	0.88 [0.84–0.93]	0.79	0.80	0.75 [0.63–0.87]	0.68	0.67
SVM	0.92 [0.88–0.96]	0.93	0.70	0.87 [0.82–0.92]	0.91	0.64	0.74 [0.63–0.86]	0.80	0.72
RF	1.00 [1.00–1.00]	1.00	1.00	0.85 [0.80–0.91]	0.92	0.53	0.67 [0.54–0.81]	0.84	0.68
Radiomics classifier									
LR	1.00 [0.99–1.00]	1.00	0.99	0.81 [0.75–0.87]	0.59	0.82	0.72 [0.59–0.84]	0.77	0.54
SVC	1.00 [1.00–1.00]	1.00	1.00	0.88 [0.83–0.92]	0.65	0.89	0.76 [0.64–0.88]	0.73	0.61
SVM	0.96 [0.94–0.98]	0.59	1.00	0.67 [0.59–0.75]	0.03	0.97	0.62 [0.48–0.75]	0.45	0.65
RF	1.00 [1.00–1.00]	1.00	1.00	0.67 [0.60–0.74]	0.14	0.91	0.57 [0.42–0.71]	0.055	1.00
Clinical-radiomics classifier									
LR	0.95 [0.92–0.98]	0.88	0.88	0.94 [0.90–0.97]	0.86	0.86	0.85 [0.75–0.94]	0.60	0.92
SVC	0.95 [0.93–0.98]	0.89	0.88	0.93 [0.88–0.97]	0.88	0.83	0.85 [0.75–0.94]	0.64	0.92
SVM	0.96 [0.94–0.99]	0.95	0.86	0.93 [0.89–0.97]	0.94	0.79	0.84 [0.74–0.94]	0.72	0.92
RF	1.00 [1.00–1.00]	1.00	1.00	0.89 [0.84–0.94]	0.96	0.62	0.82 [0.72–0.92]	0.82	0.69

In the radiomics model, the AUCs of RF, SVM, Linear SVC, and LR in the training set were 1.00, 0.95, 1.00, and 0.99, respectively. In the internal validation set, the AUCs were 0.67, 0.67, 0.87, and 0.81, respectively. The AUCs of RF, SVM, Linear SVC and LR models in the external validation set were 0.56, 0.76, 0.56, and 0.71, respectively (Table 2 and Fig. 3). The Linear SVC-based radiomics model achieved the highest AUC in both the internal validation set and the external validation set.

**Fig. 4:**
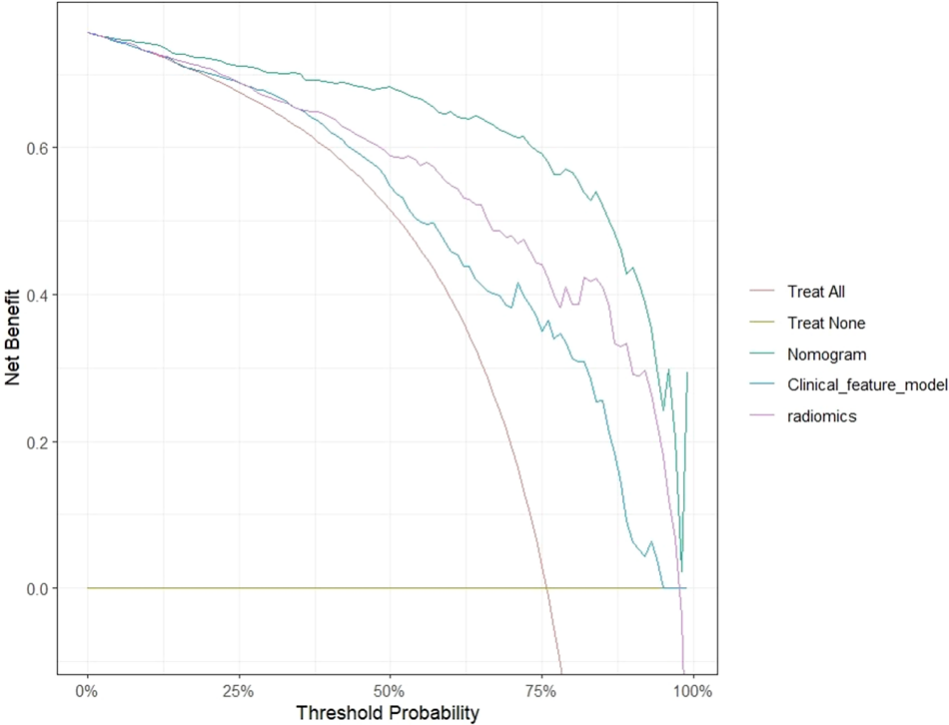
The decision curve analysis curve

**Fig. 5:**
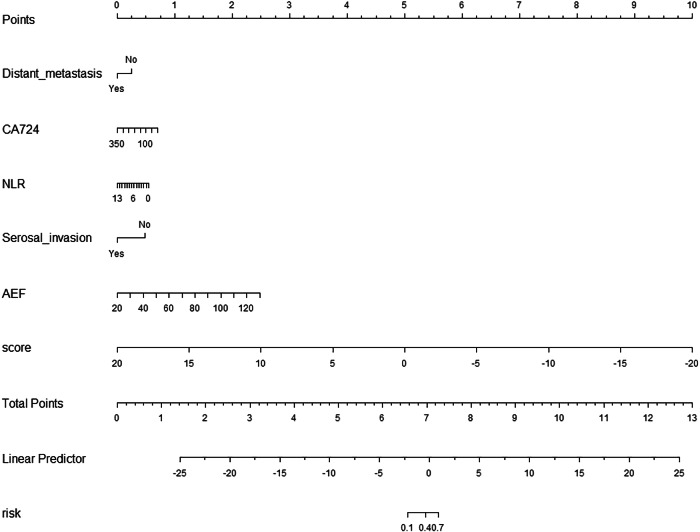
The nomogram of the clinical-radiomics model

Compared with the radiomics model and the clinical model, the clinical-radiomics model showed better performance in prediction. In the internal validation set, the LR model had the highest performance (AUC = 0.94). In the external validation set, the AUC of LR and Linear SVC were the highest, both of which were 0.846 (Table 2 and Fig. 3). The LR model achieved the best classification performance in both internal and external validation sets. The decision curve showed that the nomogram model has a better net benefit rate than the clinical model and radiomics model in further (Fig. 4). A nomogram (Fig. 5) was generated to visualize the clinical-radiomics model. The selected features and their corresponding coefficients for the radiomics, clinical, and nomogram models are summarized (Table 3).

**Table 3:** Selected features and their coefficients of the radiomics, clinic, and nomogram models

	Selected features	Coefficients
Radiomics model	wavelet-HHH_firstorder_Skewness	−0.6134
	original_firstorder_Maximum	0.9766
	original_glcm_Correlation	0.3981
	wavelet-HHH_glcm_ClusterShade	−0.4775
	wavelet-LHL_ngtdm_Busyness	1.0456
	wavelet-HHH_gldm_LargeDependenceHighGrayLevelEmphasis	0.7377
	original_firstorder_Skewness	0.7774
	wavelet-LHH_gldm_LargeDependenceLowGrayLevelEmphasis	0.2683
	wavelet-LHL_glszm_LargeAreaLowGrayLevelEmphasis	0.5925
	wavelet-LHL_firstorder_Kurtosis	−0.6837
	wavelet-LHH_firstorder_Skewness	0.2451
	wavelet-LLL_glrlm_LongRunLowGrayLevelEmphasis	1.041
	wavelet-LLL_glcm_Imc1	0.1616
	wavelet-HHL_glszm_LargeAreaHighGrayLevelEmphasis	0.6137
	wavelet-HHH_glszm_GrayLevelNonUniformityNormalized	0.3727
	wavelet-LHL_glcm_MCC	0.3268
	wavelet-LLL_glcm_Correlation	0.7617
	wavelet-HHH_firstorder_Mean	−0.3945
	wavelet-HLL_gldm_LargeDependenceHighGrayLevelEmphasis	0.7304
	wavelet-LHH_glszm_SmallAreaEmphasis	−0.7143
	wavelet-HHH_ngtdm_Strength	0.6689
	log-sigma-4-mm-3D_glrlm_RunVariance	1.4128
	log-sigma-4-mm-3D_glcm_InverseVariance	−0.6075
	log-sigma-5-mm-3D_glcm_ClusterShade	−0.8167
	original_glcm_ClusterProminence	0.5372
	wavelet-LLH_firstorder_Skewness	0.5853
	wavelet-LHH_firstorder_Kurtosis	−0.2763
	wavelet-LLH_gldm_LargeDependenceLowGrayLevelEmphasis	0.5005
	log-sigma-4-mm-3D_glcm_ClusterTendency	0.489
Clinic model	AEF	−1.384
	Distant_metastasis	−1.0332
	CA724	1.5036
	NLR	−0.5155
	Serosal invasion	−0.4388
Nomogram	Rad-score	−0.3746
	AEF	0.6042
	Distant_metastasis	−0.0527
	CA724	−0.8269
	NLR	0.606
	Serosal_invasion	−0.1646

## Discussion

In this study, we developed multiple models (clinical, radiomics and clinical-radiomics models) based on clinical and radiomics features before treatment for early prediction of PD-1 inhibitor combined chemotherapy response in GC. We also compared the performance of various machine learning classification algorithms (RF, LR, SVM, Linear SVC). Among these models, the clinical-radiomics model, which integrates both clinical and imaging features, demonstrated better prediction performance compared to the clinical model or radiomics model. Furthermore, the clinical-radiomics model integrated with the LR algorithm exhibited consistently high prediction ability in both the internal validation set and the external validation set, which was superior to other machine learning methods.

Currently, limited studies have explored the use of imaging to evaluate the efficacy of immunotherapy combined with chemotherapy in GC. Liang et al [24] retrospectively collected the clinicopathological risk factors and imaging characteristics data of 87 patients in a single center and established a radiomics nomogram. The AUCs were 0.86 and 0.78 in the training set and validation set, respectively; however, its small sample size compromised generalizability. To address this limitation, we incorporated a larger dataset with independent external validation sequences to enhance the generalizability of our model. The clinical-radiomics nomogram we propose is a biomarker with potential clinical application value. Wang et al [25] used data from 249 patients to evaluate the synergistic effect of multimodal data (clinical and imaging data) in predicting the immune response pattern of GC. Their findings align with our conclusion that the clinical-radiomics model incorporating clinical information had a higher predictive ability than the radiomics model. This may be because single-omics data cannot capture the complexity of immunotherapy response [26–29]. Compared to Wang’s study [25], our model achieved a higher AUC (0.85 vs. 0.81) in the external validation set. The calculation of machine learning radiomics features is influenced by various changes, including differences in imaging centers, CT scanner models and scanning parameters [30–32]. To reduce the impact of data heterogeneity on the predictive ability of the model, we used preprocessing techniques of normalization, standardization, and resampling [33–35].

A total of 19 CT radiomics features were selected, including one first-order feature, two Gaussian transform features, and 17 wavelet features. First-order features, which encompass morphological features and histogram features, are directly calculated based on the original image pixel gray distribution. Fewer first-order features were screened, possibly due to the peristalsis of the gastrointestinal cavity organs, whose morphological features are less fixed. Wavelet features capture high-order information and spatial and frequency component analysis on multiple scales. The features after wavelet changes can retain the image sharpness, which is conducive to displaying the original image information [36, 37]. Collectively, the first order, Gaussian transform and wavelet features of GC CT before treatment reflect the heterogeneity of the tumor from different feature dimensions.

NLR, CA72-4, distant metastasis, serosal infiltration (visceral peritoneum), and AEF are associated with immunotherapy response in GC patients, each of which has been validated as a predictive biomarker. NLR is positively correlated with the density of depleted cd8t cells in the tumor microenvironment that reflects the attenuation of T cell function [38]. High expression of CA72-4 usually indicates AGC stage and poor prognosis. CA72-4 can be used to predict the efficacy of neoadjuvant chemotherapy, palliative chemotherapy and immunosuppressive therapy in patients with GC [39, 40]. Tumor cells and the tumor immune microenvironment (TME) in metastatic lesions may systemically modulate the immune microenvironment of distant primary tumor tissues via circulatory mechanisms [41]. Consequently, tumor metastasis status affects the efficacy of immunotherapy [42]. Furthermore, the presence or absence of the serosal infiltration (visceral peritoneum) and AEF are also important factors in predicting treatment response.

In the comparison of multiple machine learning methods, we found that in the clinical model and clinical-radiomics model, the LR method achieved the highest AUC (0.89 in the internal validation set, 0.76 in the external validation set; 0.94 in the internal validation set, 0.85 in the external validation set). The Linear SVC radiomics model achieved the best AUC (0.88 in the internal validation set and 0.76 in the external validation set) among the radiomics models. These findings suggest that the clinical-radiomics models based on LR may provide a more effective approach for clinical decision making and hierarchical management of GC patients after immunotherapy. Logistic regression, a supervised machine learning method, builds relationships between input features and output probabilities by fitting a sigmoid curve to a dataset. Compared to other machine learning models, LR offers greater interpretability and presents advantages in clinical applications [33].

Notwithstanding, our study has several limitations. Firstly, as a retrospective study, it is subject to potential recall bias. In addition, although our study is larger than those used in previous studies, online data sets and data from more centers can be added in future studies to more fully validate the generalization ability of the model. Moreover, despite applying preprocessing techniques such as resampling, and normalization, the performance of the model may still be affected by variations in data sources and CT manufacturers.

## Conclusions

In summary, we compared the prediction performance of various machine learning classifiers and demonstrated the feasibility of clinical-radiomics models in predicting the internal and external efficacy of PD-1 inhibitors combined with chemotherapy in AGC patients at an early stage. The nomogram model combining clinical risk factors and radiomics signatures proposed in this study can conveniently provide individualized treatment strategies for clinicians before treatment.

## Supplementary information

ELECTRONIC SUPPLEMENTARY MATERIAL

## Data Availability

The datasets used and/or analyzed during the current study are available from the corresponding author on reasonable request.

## Abbreviations

AEF: Arterial enhancement fraction
AGC: Advanced gastric cancer
AUC: The area under the receiver operating characteristic curve
CR: Complete response
GC: Gastric cancer
ICC: Intraclass correlation coefficients
Linear SVC: Linear support vector classification
LR: Logistic regression
NLR: Neutrophil to lymphocyte ratio
PD: Progressive disease
PD-1: Programmed cell death-1
PD-L1: Programmed death-ligand 1
PR: Partial response
RECIST 1.1: The Response Evaluation Criteria in Solid Tumors 1.1
RF: Random forest
ROC: Receiver operating characteristic
SD: Stable disease
SVM: Support vector machine
