# Supplementary material for: Multimodal CT radiomics predicts PD-1 inhibitor efficacy in advanced gastric cancer: a two-center validation study
Source: Insights Imaging. 2025 Oct 31;16:235. doi: 10.1186/s13244-025-02096-1 (PMC12575892; doi:10.1186/s13244-025-02096-1)
Supplement: Supplementary file 1 — ELECTRONIC SUPPLEMENTARY MATERIAL [file 13244_2025_2096_MOESM1_ESM.pdf]

Multimodal CT Radiomics Predicts PD-1 Inhibitor Efficacy in Advanced Gastric Cancer: A Two-Center Validation Study

ELECTRONIC SUPPLEMENTAL MATERIAL

Table S1. CT scan protocols

Modality	CT				
**Scanner model**	SOMATOM Force	SOMATOM Definition Flash	SOMATOM Drive	Aquilion ONE	Ingenuity CT
**Manufacturer**	SIEMENS	SIEMENS	SIEMENS	TOSHIBA	Philips
**Tube voltage (kV)**	90	120	120	120	120
**Tube current**	245	256	411	100	240
**Matrix**	512×512	512×512	512×512	512×512	512×512
**Slice thickness (mm)**	1	1	1	1	1
**Exposure time (ms)**	500	500	330	500	415

kV, kilovolt; mm, millimeter; ms, millisecond
